# The lab-field continuum in conservation physiology research: leveraging multiple approaches to inform policy and practice

**DOI:** 10.1093/conphys/coaf063

**Published:** 2025-09-02

**Authors:** Sandra A Binning, Kerri Lynn Ackerly, Steven J Cooke, Marco Fusi, Daniel F Gomez Isaza, Emily A Hardison, Sidney Martin, Amelia Munson, Mar Pineda, Gail D Schwieterman, Martin Reichard, Andrea Rummel, Tamzin A Blewett

**Affiliations:** Département de Sciences Biologiques, Université de Montréal, 1375 Ave. Thérèse-Lavoie-Roux, Montréal, QC H2V 0B3, Canada; The University of Texas at Austin Marine Science Institute, Department of Marine Science, 750 Channel View Drive, Port Aransas, TX 78373 USA; Department of Biology, Carleton University, 1125 Colonel By Drive, Ottawa, ON K1S 5B6, Canada; Dove Marine Laboratory, School of Natural and Environmental Sciences, Newcastle University, Newcastle NE1 7RU, UK; Australian Institute of Marine Science, Indian Ocean Marine Research Centre, 64 Fairway, Crawley 6009, WA, Australia; Department of Biological Sciences, University of Pittsburgh, 4249 Fifth Avenue, Pittsburgh, PA 15260, USA; Department of Biological Sciences, University of Alberta, 11335 Saskatchewan Dr. NW, Edmonton, AB T6G 2E9, Canada; Department of Wildlife, Fish, and Environmental Studies, Swedish University of Agricultural Sciences, Skogsmarksgränd 17, Umeå, 907 36, Sweden; School of Biodiversity, One Health & Veterinary Medicine, University of Glasgow, Science Way, Glasgow, G12 8QQ, UK; School of Marine Sciences and the Maine Agricultural and Forest Experiment Station, University of Maine, 168 College Ave, Orono, ME 04469, USA; Institute of Vertebrate Biology, Czech Academy of Sciences, Květná 8, 603 00 Brno, Czech Republic; Department of Botany and Zoology, Faculty of Science, Masaryk University, Kotlářská 2, 611 37 Brno, Czech Republic; Department of Ecology and Vertebrate Zoology, University of Lodz, Narutowicza 68, 90-136 Lodz, Poland; Department of BioSciences, Rice University, 6100 Main St, Houston, TX 77005, USA; Department of Biological Sciences, University of Alberta, 11335 Saskatchewan Dr. NW, Edmonton, AB T6G 2E9, Canada

**Keywords:** Accessibility, behavioural ecology, knowledge-brokers, mesocosm experiments, modelling, partner-integrated work flow, video tracking

## Abstract

In the field of conservation physiology, there is often a trade off between conducting research in controlled laboratory settings or in inherently variable field environments. However, this belief sets up a false dichotomy where laboratory experiments are perceived as providing precise, mechanistic understanding with low variability at the cost of environmental realism while field studies are ecologically relevant but criticized for generating inconsistent evidence that is difficult to interpret and replicate. Despite the perceived binary view, these approaches are not in opposition to one another, but rather form a continuum along increasing ecological complexity. Here, we argue that it is possible to mindfully and purposefully design studies and develop integrative collaborations in conservation physiology that span the lab-field continuum to address pressing environmentally-relevant questions that can be used to inform policy and practice. We first outline the advantages and disadvantages of different approaches to knowledge generation. We then highlight ways to bridge the lab-field divide though leveraging the advantages provided by different approaches to build a more comprehensive understanding of the natural world, including how recent technological advances can help connect lab- and field-based research. Next, we discuss the importance of partnership and collaboration across sectors for informing our understanding of ecological patterns and physiological processes. Finally, we reflect on how to best translate physiological research into action and the reciprocal role that environmental practitioners can have in driving research questions in conservation physiology.

## Introduction

A common aim for experimental biologists working on free-living macrorganisms is to better understand the principles shaping the natural world (Weber, 2004; Stillman *et al.,* 2011). In conservation physiology, this knowledge is often reached through hypothesis-driven quantitative and qualitative research typically conducted in the laboratory (i.e. purpose-built controlled settings designed for maximum control over environmental variables) and/or field (i.e. natural settings with little or no control over environmental variables). Indeed, laboratory and field studies are often presented as either-or approaches, each offering distinct advantages and challenges for studying physiological and ecological processes (Calisi and Bentley, 2009). The high degree of control provided by laboratory experiments facilitates measurement precision, promotes an understanding of causal relationships and mechanisms and reduces the confounding effects of environmental and/or individual variation (Diamond, 1986; Calisi and Bentley, 2009). However, the resulting oversimplification of biological processes raises questions about the environmental relevance of findings and applicability to conservation practice (Diamond, 1986; Cooke *et al.,* 2017). Field studies, on the other hand, align organismal responses with natural environmental fluctuations, often providing conservation practitioners with more relevant information to base policies and interventions (Cooke and O’Connor, 2010; Madliger *et al.,* 2021). Yet, the inherent unpredictability and variability of these studies hampers replicability and, thus, an easily interpretable, mechanistic understanding of physiological processes (Costa and Sinervo, 2004). Many factors may determine what approach is selected by a researcher for a given study including issues of access, ethics, permits, financial costs and safety, among others. Regardless of how decisions are made, conservation physiologists may feel they must make a dichotomous choice between the ecologically-artificial lab or the fickle field knowing that either choice will result in intense scrutiny from researchers firmly rooted in the other ‘camp’.

**Figure 1:**
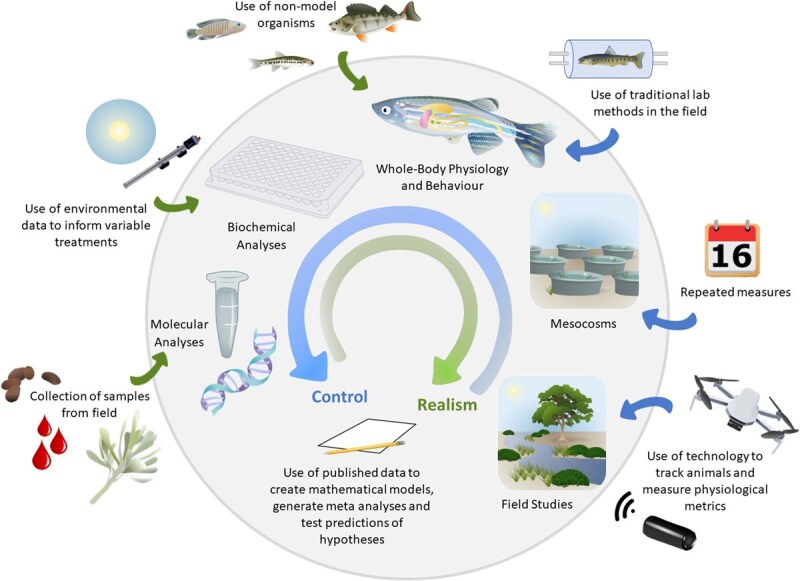
An illustrative representation of the relationship between control and realism in conservation physiology. The outside circular arrow indicates increasing control and internal circular arrow indicates increasing realism. Studies within the grey circle indicate where traditional types of studies fall on this continuum. Outside of the circle are proposed ways of increasing control or realism across the continuum. Figure artwork by A.M.

For decades, ecologists have recognized that laboratory and field experiments form a continuum of approaches depending on the degree to which the physical and/or biotic environment is regulated and/or manipulated by the researcher (Diamond, 1986; Sasaki *et al.,* 2025). This flexibility has allowed the tools and techniques used in ecological research to evolve and expand across the years giving ecology a central place in conservation research (Anderson *et al.,* 2021). As conservation physiology researchers, we also advocate for a more integrative approach that positions laboratory and field studies as existing along a continuum of research practices across increasing ecological complexity (Fig. 1). In this perspective article, we first explore the strengths and limitations of laboratory and field approaches that are traditionally used by conservation physiologists. Next, we discuss how leveraging the advantages of these different methods can help bridge the lab-field divide and lead to a more holistic understanding of the natural world, highlighting how recent technological advancements can break down the boundaries between lab and field-based research in our discipline. We then emphasize the importance of cross-sector collaboration and partnerships in ensuring the relevance of ecological patterns and physiological processes under study recognizing that the way research is conducted is as important as the findings. Finally, we reflect on how to effectively translate experimental research along the lab-field continuum into policy action and the reciprocal influence that environmental practitioners can have in shaping research questions in fundamental and applied animal physiology.

### Pros and cons of laboratory and field experiments in conservation physiology research

Selecting a research question is the first challenge in study design, followed by understanding the appropriate tools to answer the question (Diamond, 1986). Indeed, the conservation physiology ‘toolbox’ is diverse with some approaches better suited for one set of study conditions than others (Madliger *et al.,* 2018). Here, we summarize some of the common benefits and drawbacks of conducting research in different environments with the goal of illustrating the utility each approach can provide (Table 1). We also want to emphasize that there is no ‘perfect’ approach. For example, primatologists have long recognized that observer effects can significantly impact primate behaviour in both lab and field settings (Caine, 1990; Nowak *et al.,* 2014; Piel *et al.,* 2022). However, careful and intentional consideration of experimental designs and data collection methods can reduce issues impacting research outcomes.

**Table 1:** The advantages and disadvantages of laboratory and field-based approaches

	**Advantages**	**Disadvantages**
**Laboratory approaches**	**Scientific**	
	Organism’s environment is tightly controlled and monitored (limits confounding factors)	Fail to replicate environmental complexities
	Establish cause and effect relationships	Short durations (often data represents a ‘snapshot in time’)
	Easier to replicate	Involve the transfer of organisms to artificial settings
	Availability and access to specialized tools and technology (e.g. microscopes, PCR machines, incubators).	Incorporating environmental complexity can reduce statistical power to detect differences among treatments
	Can provide proof-of-concept	Favours research on model species
	Easier and less costly to test treatments prior to field-based testing	Restricted to smaller sized experiments
		Restricted to smaller sized organisms (space constrains limit physical size of test organisms)
		Loss of the natural social context of research organisms (can induce additional stress in test subjects)
	**Economic**	
	Reduced travel needs (experiments are often based at home institutions which minimizes costs associated with travel and accommodation on-site)	Resource intensive (specialized scientific equipment can be expensive. Often requires access to space and technical staff.
	**Social**	
		Ethical concerns of subjecting animals to unrealistic laboratory conditions, treatment, isolation and potential harm.
	Facilitates work-life balance allowing researchers to reside in their usual place of residence (when laboratories are based at home institutions)	
**Field approaches**	**Scientific**	
	Organisms exposed to biotic and abiotic interactions reflecting the complexity and variability of real-world ecosystems	Lack of control which can introduce experimental confounds or additional unmeasured variables
	Long term data possible (e.g. remote monitoring such as biotelemetry, weather stations).	Experimental logistics can be complex (e.g. requires additional planning for transport, accommodation, equipment and access to field locations)
	Improved environmental relevance in policy development	Data is variable and difficult to replicate
	Reflects natural population variability (results can be generalized to the population-level	Access to organisms is not consistent
	Reduced stress by limiting interaction with researchers and the lab environment	High inter-individual variation
	**Economic**	
		Time and resource intensive which can restrict the number of sampling locations or duration of a field-based project
		Travel costs can be high especially when accessing remote locations

(Continued)

**Table 1:** Continued

	**Advantages**	**Disadvantages**
	**Social**	
	Interactions with local groups fosters trust and can promote co-creation	Safety risks especially when working in remote locations where access to health services may be difficult
		Ethical concerns of disrupting the natural environment, especially when conducting field work on sites or species of local cultural importance

In general, the laboratory environment is often most appropriate for questions involving ‘bench work’ techniques where specialized equipment can be installed, often close to where organisms are being held, and environmental conditions can be more carefully controlled and monitored. The laboratory environment can also be appropriate for questions aimed at examining physiological processes and mechanisms at the molecular, cellular and organismal levels (e.g. environmental acclimation, toxicant exposure, whole-organism and cellular metabolism, maximum performance capacity; Calisi and Bentley, 2009; Nelson, 2016). Laboratory studies can also be used to pilot approaches before being applied to more costly field settings. For example, measures of fish swimming performance and behaviour derived from the lab have been used to inform conservation strategies in the wild (Poletto *et al.,* 2015; Rodgers *et al.,* 2017; Watson *et al.,* 2018). Further, although accessibility barriers exist in all research settings, field work can present heightened risks and distinct accessibility challenges which require additional efforts to overcome (see Rudzki and Kohl, 2023).

Studies conducted in laboratory settings do have constraints, which can limit their real-world applicability (Rose *et al.,* 2020). They often occur over short time durations, providing only a snap-shot in time, and involve bringing live organisms into artificial settings, which can fundamentally alter their physiology and behaviour (Turko *et al.,* 2023). Although there have been increasing attempts to replicate environmental complexity in the lab in recent years (Gerhard *et al.,* 2023; Rodgers and Gomez Isaza, 2023; Orr *et al.,* 2024), these studies can rapidly become overwhelming in size and have reduced statistical power to detect differences among treatments (Table 1). Organismal size (e.g. difficult to bring a whale shark (*Rhincodon typus*), African elephant (*Loxodonta africana*), or giant sequoia (*Sequoiadendron giganteum*) into the lab) or the rarity/conservation status of a given species may also limit the extent to which organisms can be subject to experiments in a captive laboratory environment. Also, laboratory experiments typically test individuals in isolation regardless of the species’ natural social context. In the case of animal research, testing animals in isolation can produce different results than if animals were tested in groups making some lab-generated results even further removed from ecological reality (e.g. Burggren *et al.,* 2017; Overduin *et al.,* 2025).

Field studies inherently integrate aspects of abiotic (e.g. temperature, salinity, oxygen, humidity, pH, landscape heterogeneity) and biotic (e.g. community composition, species interactions, disease, life-history) variation, which can be difficult or impossible to replicate in laboratory settings (Table 1). Insights gained from field studies are also crucial for parameterizing laboratory studies such that experimental conditions replicated in the laboratory are realistic. However, the availability of time, personnel and funding may restrict the number of sampling locations or duration of a field-based project, limiting its scope. While technological advances have greatly improved researchers’ ability to record a diverse suite of physiological parameters (Calisi and Bentley, 2009), access to experimental organisms in the field can be challenging, particularly if there is interest in repeated sampling over time. Individual histories are often unknown, and field studies must contend with high levels of inter-individual variation in traits, including condition, health, nutritional status and performance (Simonis *et al.,* 2025). Although this heterogeneity represents real and meaningful biological variation, too many unexplained and unaccounted for differences among individuals may mask relationships when comparing group means (Williams, 2008).

 In both field and laboratory settings, researchers from marginalized groups often face distinct and intersecting systemic barriers to accessibility, safety, and inclusion (Clancy *et al.,* 2014; Viglione, 2020; Rudzki *et al.,* 2022; Coon *et al.,* 2023; Rudzki and Kohl, 2023; Verble *et al.,* 2023). Across the continuum of research approaches, there is a continued need for institutions, field stations and research teams to to implement measures that actively dismantle systemic barriers and ensure equitable and safe environments (Gin *et al.,* 2022; Ramírez-Castañeda *et al.,* 2022; Rudzki *et al.,* 2022; Rudzki and Kohl, 2023).

### Bridging the lab-field divide

There is no one-size-fits-all approach to designing studies in conservation physiology. Although mismatches between lab and field studies can be helpful in understanding mechanistic drivers of observed responses (Calisi and Bentley, 2009), designing effective conservation strategies requires robust and predictable outcomes. In most cases, integrating elements across the laboratory-field continuum can foster a stronger balance between controlled experiments and real-world environmental considerations. For example, mesocosms, partly enclosed indoor or outdoor experimental units, have been used as a tool in ecological and environmental studies for decades (Odum, 1984; Sasaki *et al.,* 2025; Fig. 1). Such integrative studies can offer broad and fundamental insights into factors such as the interaction of multiple environmental variables on physiological responses (e.g. generating dose response curves, understanding cumulative effects), the physiological changes associated with short- and long-term acclimation to standardized conditions, the representativeness of standard laboratory model species as stand-ins for their wild counterparts, and paths towards scaling-up from individual to community- and ecosystem-level impacts (Fig. 1; Odum, 1984; Calisi and Bentley, 2009; Newman *et al.,* 2015; Bergman *et al.,* 2019; Ames *et al.,* 2020; Turko *et al.,* 2023). For instance, in studies of thermal tolerance in mangrove crabs (*Parasesarma guttatum, Tubuca urvillei*), field observations helped frame and contextualize laboratory thermal experiments (e.g. Fusi *et al*., 2014). Similarly, Marshall *et al.* (2013) integrated behavioural field data with physiological measurements to assess climate vulnerability in high-rocky-shore snails (*Echinolittorina malaccana*).

One way to bridge the lab/field divide is to enhance environmental relevance and complexity in controlled laboratory settings. For example, incrementally introducing environmental complexity into laboratory systems combined with knowledge of physiological mechanisms has improved the prediction of metal toxicity in aquatic organisms (e.g. Hogstrand and Wood, 1998; Blewett *et al.,* 2018; Mebane, 2023). Similarly, incorporating fluctuating exposures to environmental conditions that vary naturally in the field have allowed researchers working in laboratory studies to identify distinct physiological responses to constant, regular- and/or irregular changes in the environments, providing better insights into the likely outcomes of extreme environmental variability in nature (Matsubara, 2018; Nancollas and Todgham, 2022). While these studies often focus on localized regions, the potential for collecting field data to inform laboratory work is rapidly expanding due to advancements in climate and environmental monitoring. High-resolution datasets from urban observatories, such as the Newcastle Urban Observatory (https://newcastle.urbanobservatory.ac.uk), now offer unprecedented access to fine-scale environmental conditions. In parallel, large-scale coordinated initiatives like Tara Oceans (https://fondationtaraocean.org/en/home/) have created globally distributed field datasets encompassing environmental, biological and genomic information. The Tara Oceans project, in particular, has inspired a prolific body of peer-reviewed literature (see Sunagawa et al.*,* 2020). These datasets have been pivotal for the development and testing of new ecophysiological hypotheses in controlled laboratory settings.

Building on these principles, mesocosm studies can be designed in terrestrial, aqauatic, or semi-aquatic systems to allow some degree of control over biotic and/or abiotic parameters of interest while allowing more realistic fluctuations that better approximate natural environments (e.g. Sharma *et al.,* 2021; Nespolo *et al.,* 2022, Brisson *et al.,* 2024; Fig. 1). Mesocosms can vary in size (e.g. several hectares, Lin *et al.,* 1999; thousands of litres, Ussery *et al.,* 2024; microcosms, Brisson *et al.,* 2024). They can be monitored over long (e.g. months-years, Pace *et al.,* 2019) or short (e.g. days; Orlikowska *et al.,* 2015) timeframes. Increasingly, mesocosms are instrumented with data logging devices permitting real-time recordings of fluctuations, while also enhancing experimental designs of in-lab mesocosm experiments (e.g. Pansch and Hiebenthal, 2019). Although they often cannot replicate the full complexity of natural ecosystems, they nevertheless can help address some of the shortcomings of more traditional lab approaches (Table 1).

Field studies take environmental realism one step further than mesocosm studies. Yet, they may still benefit from the integration of some traditionally lab-based approaches. In the field, a key challenge is identifying the driving (causal) factors of a given physiological response. Although difficult to achieve, a growing group of researchers is attempting to understand the physical, chemical and/or biological components of the environment at the time the physiological parameter is measured to better understand the impacts of multiple stressors on organisms (e.g. Todgham and Stillman, 2013). Repeated measurements on the same individuals and/or in the same locations over time facilitates the identification of the environmental variables that most meaningfully induce physiological or behavioural change (Williams, 2008; Roche *et al.,* 2016). For example, estimates of metabolic rates made in the field helped to demonstrate that circadian rhythms play an important role in shaping responses of rainbow trout (*Oncorhynchus mykiss*) to environmental temperature, a relationship that would have been difficult to uncover if similar measures had taken place solely in a laboratory setting (Briggs and Post, 1997).

Another way to connect lab and field studies is through the use of published data to inform mathematical modelling. Empirical data, particularly data that can be used to identify the mechanisms by which specific environmental stressors affect specific a physiological pathway, allow for the development of predictive models (Fig. 1). Such models can be used to better understand and predict how environmental variation and anthropogenic change will impact wild populations (Glover, 2018). Modelling approaches can help generate hypotheses, predict population and community level responses to environmental change, assess risk of vulnerable populations to stressors, forecast species distributions and abundance and better understand disease dynamics and bioenergetics (Rohr *et al.,* 2013; Evans *et al.,* 2015). Previously published data, from both field and lab studies, can also be used to test hypotheses across a range of species and settings (e.g. Khaliq *et al.,* 2014). While most conservation models still lack physiological information (Evans *et al.,* 2015; Urban *et al.,* 2016), new approaches increasingly allow the integration of ecophysiological traits into modelling frameworks (e.g. Gamliel *et al.,* 2020). In all cases, model validation is critical to assess their appropriateness, relevance and accuracy (Garman *et al.,* 2020).

Despite their advantages, all models are only as useful as the data they rely on; an over reliance on lab-based studies, for example, without validation and integration with field-based work can lead to predictions that have many of the same drawbacks as a single lab-based study. Similarly, modelling approaches rely on empiricists to continue generating robust data on which to base and test their predictions. An over-reliance on modelling approaches, which are often perceived to be more cost-effective than empirical research, runs the risk of disincentivizing researchers from collecting primary data, which will ultimately limit the usefulness of predictive models. The integration of modelling and field/lab-based techniques is hampered by a lack of cross-disciplinary expertise: conservation physiologists typically do not have expertise in laboratory, field and quantitative modelling approaches. These challenges can be overcome through cross-disciplinary and multi-institutional collaborative research (Roche *et al.,* 2022; Nakagawa *et al.,* 2025; see section: What can we gain through partnership and collaboration?). Additionally, the community would benefit from more open-source resources and training opportunities to learn how to use physiology data to inform conservation-focused models and vice-versa.

### How can we leverage technological advances to bridge gaps?

Leveraging technological advances can significantly improve experimental design, measurement accuracy, the application of results to real-world scenarios and bring techniques that were traditionally tied to the lab into the field (Fig. 1; Fausch *et al.,* 2002; Schwartz *et al.,* 2004; Binning *et al.,* 2022). For example, uncrewed aerial vehicle (a.k.a. drones) technology is increasingly used to monitor plant physiology and health in both cultivated and uncultivated settings (Gano *et al.,* 2024). Similarly, advances in computer vision approaches now enable the automatic tracking of animal groups using drone footage, which can provide data on wild animal behaviour that was previously only possible to obtain via laboratory studies (Cooke, 2008; Koger *et al.,* 2023). Technological advances in biologging and telemetry are also further expanding the size and types of animals that can be studied and the speed and type of data gathered. These advances expand the potential for field studies to examine not only animal movement, but also energetics, kinematics, health and behaviour through these techniques (e.g. Signer *et al.,* 2010; Kays *et al.,* 2015; Beardsworth *et al.,* 2022; Binning *et al.,* 2022). There are also now sensors that can be incorporated into electronic tags to measure a range of physiological health indicators (e.g. blood glucose, heart rate, body temperature) and limb movements in free-living animals (Neethirajan, 2017; Binning *et al.,* 2022), which were previously limited to lab-based studies. These technological advances can also be used in all study settings as a way of collecting behavioural and physiological data without the confounding presence of human observers (e.g. Brown *et al.,* 2013; Piel *et al.,* 2022). Looking forward, advances in human physiology research have demonstrated that virtual reality may be a useful tool that can also help bridge the gap between environmental complexity and laboratory conditions (Weibel *et al.,* 2018; Halbig and Latoschik, 2021). These technologies are increasingly being adapted to for use on non-human species such as insects and fishes (e.g. Fry *et al.,* 2008; Huang *et al.,* 2020; Vidal *et al.,* 2023). The future development of this technology across a broader swath of taxa in a field-based context is a fascinating area of future development.

Laboratory-based projects can also be made more ecologically relevant with technology. Indeed, advances in material science have improved the capacity of environmental sensors, making it easier to retrieve environmental data at ecologically relevant scales, which can then be used to inform lab-based treatments (Giomi *et al.,* 2023). Increasing automation of experimental set-ups for collecting behavioural and physiological data (e.g. Couzin and Heins, 2023; Ajuwon et al., 2024; Ern and Jutfelt, 2024; Lucks *et al.,* 2024) can also help simplify logistics, which may facilitate increasingly complex experimental designs to test the impact of more ecologically relevant conditions. Similarly, virtual reality can be used to study the impact of standardized stimuli on an organism combining the precision of lab-based measures with more ecologically relevant conditions (Vidal *et al.,* 2023). Special built technology may also help expand the use of mesocosms in lab-based work. The SMART-BARN, for instance, is a large arena containing a motion capture system, video cameras, acoustic sensors and multiple remote-controlled interactive units allowing for the simultaneous recording of multiple data streams for groups of animals from diverse taxa including insects, birds and mammals (Nagy *et al.,* 2023). Technology also facilitates the development of ‘do it yourself’ tools allowing researchers to develop tailor-made solutions for a diversity of research settings. The Raspberry Pi, for example, has broad capacities with a relatively small price tag making it ideal for applications including nest-box recording, wildlife camera-trapping, plant phenotyping, underwater video monitoring, and closed-loop behavioural experiments (Jolles, 2021). 3D-printers have also become cheaper and more accessible, allowing researchers to custom-make tools for behaviour and physiology research applicable to a diversity of settings (Behm *et al.,* 2018; Walker and Humphries, 2019).

While there is great potential for technological advances to move research forward, it also introduces new challenges. Many new technologies rely on high efficiency batteries, which limit usability (Folea and Mois, 2020). The cost of integrating new technologies into experimental design also remains prohibitively high for many researchers, exacerbating existing inequalities among research groups both within and across geographic regions. Additionally, the unprecedented level of data collection facilitated by technology poses a challenge to the transmission, storage, use and analyses of these data. Advances in computer technologies have streamlined this process and led to more open-source databases, but ways to democratize data remains an on-going consideration (Dessimoz and Thomas, 2024). Further, existing machine learning models often rely on biased data representing specific geographic regions and curating big data sets while ensuring proper credit is attributed can be challenging (Tuia *et al.,* 2022). New quality control methods are also needed, as is a careful consideration of the financial and environmental costs of new technologies (Tuia *et al.,* 2022). Although different strategies for effectively and ethically integrating new technologies into research programs exist (Marvin *et al.,* 2016), even the most promising new technology cannot make up for a poorly designed experiment. As such, researchers working across all facets of conservation physiology must continue to leverage the benefits of new technology while remembering that the most powerful tool is an individual’s ability to think critically about their research. Ultimately, no amount of technology can fully replace traditional, observation-based knowledge about ecosystems, and mindful integration of these two approaches is likely to yield the most promising outcomes (e.g. Finerty *et al.,* 2024).

### What can we gain through partnership and collaboration?

An iterative process that encourages collaboration across researchers spanning the field-lab continuum ensures that, when appropriate, laboratory-based research remains environmentally relevant and applicable to more natural settings (Cooke *et al.,* 2014). Similarly, collaboration across these research approaches can improve relevance of field research by bolstering conclusions with mechanistic data. This collaborative approach also facilitates the development of adaptive strategies for modelling, which is particularly useful of informing conservation actions to adapt to climate change. Large collective efforts are often needed to optimize the exploration of multidimensional experimental space (Box 1). While it may be unreasonable to expect individual researchers or even solo-PI led teams to possess the means and expertise to conduct research in both lab, field and *in silica* realms, collaborative efforts among teams can also enhance research impacts.

Box 1:
**Cross-disciplinary collaboration in Action: Scientific Committee on Oceanic Research**

One successful example of large-scale interdisciplinary collaboration is the international Scientific Committee on Oceanic Research (SCOR, 2025), which was established to bring together teams of researchers to answer large-scale questions related to ocean sciences. SCOR supports international working groups to advance ocean science by promoting international cooperation in planning and conducting oceanographic research as well as addressing interdisciplinary and multidisciplinary ocean issues. For instance, the Changing Ocean Biological Systems Working Group (COBS; WG 149), brought together research with a diverse range of expertise in physiology, ecology and evolutionary biology to assess broad questions about the impacts of climate change on ocean biota. The goal of the COBS working group is to understand how marine life responds to concurrent changes in oceanic conditions due to climate change. These concurrent changes, known as multiple environmental drivers, include factors such as temperature fluctuations, ocean acidification, deoxygenation and nutrient shifts. COBS aims to develop methodologies to assess the cumulative effects of these drivers on marine organisms and ecosystem, thus moving beyond single-factor studies to experiments that consider multiple interacting environmental drivers. This approach seeks to provide a more comprehensive understanding of how marine organisms and ecosystems respond to the complex nature of climate change. The group has created resources like the MEDDLE (Multiple Environmental Driver Design Lab for Experiments) platform, which offers guidance and tools for researchers designing multi-driver experiments. This includes a three-step guide to help identify relevant drivers, design experiments and finalize methodologies. COBS also trains scientists in multi-driver research through various programs, including PhD and MSc courses, summer schools, conferences and online courses. This effort aims to equip the next generation of marine scientists with the skills needed to conduct rigorous and relevant research in the context of a changing ocean. Handbooks supporting best practices for multiple-driver marine research, are available to download as well as several peer-reviewed papers to help unify methodology and experimental approaches in order to reproduce environmentally relevant conditions in the laboratory (Boyd *et al.,* 2018). As a result of the collaborative network derived from this initiative, researchers can pool resources and data from multiple labs, increasing sample sizes and variability in experimental conditions.

**Figure 2:**
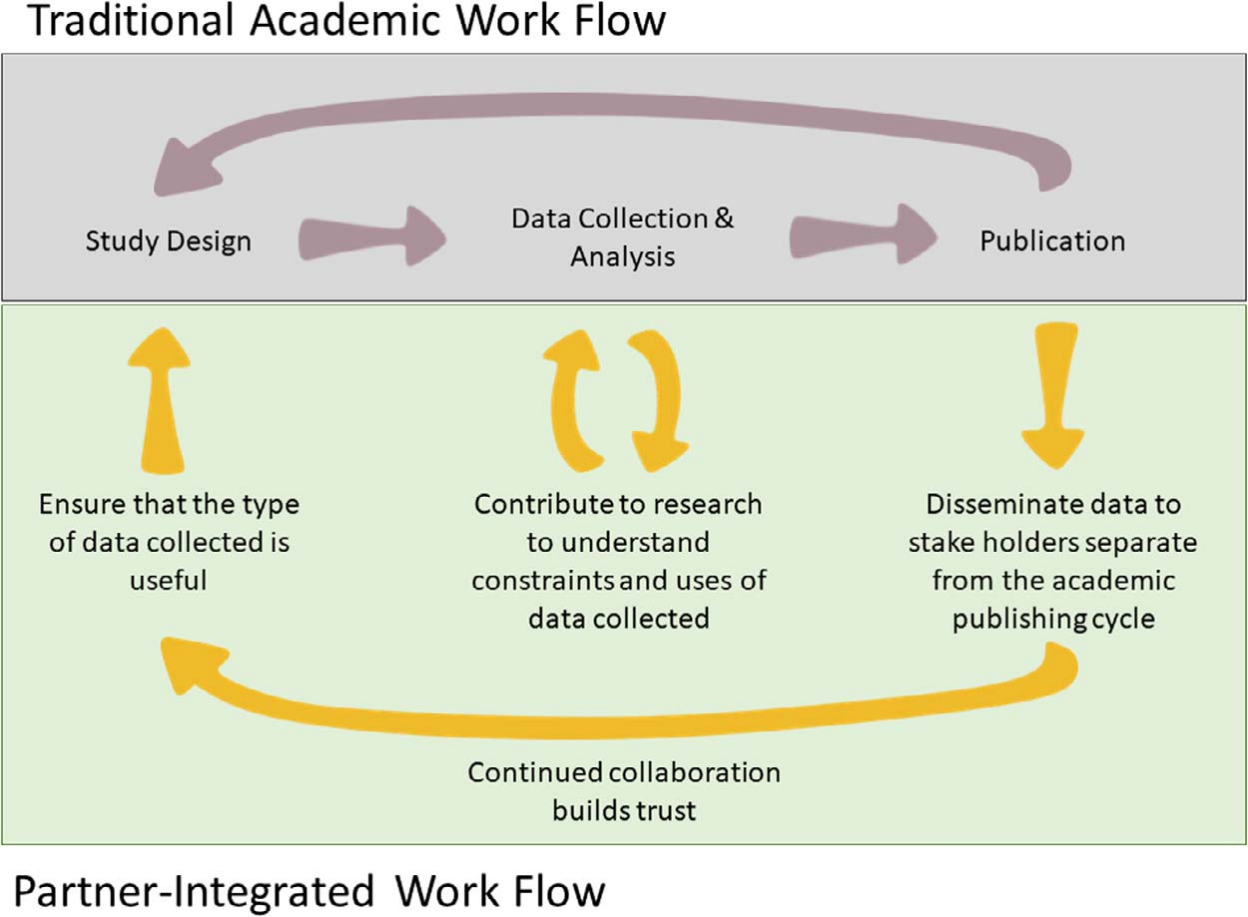
Illustration of ways that the traditional academic work flow (top panel) can be expanded to integrate feedback from knowledge users in a partner-integrated works flow (bottom panel)), including managers, community members, policy makers and others. Arrows in the bottom panel indicate the flow of benefits (e.g. study design benefits from the input of knowledge users to make research more useful).

An additional, and often overlooked, way to move scientific knowledge into the action sphere and make research more relevant to society is through collaborations with and among non-academic partners. The most effective of these collaborations embrace a co-production model where knowledge generators, knowledge users and other actors work together to develop research questions, secure funding, conduct the research and interpret findings while incorporating diverse perspectives (Fig. 2; Beier *et al.,* 2017; Norström *et al.,* 2020). Co-production requires high levels of trust among all parties, mutual respect and time (which is inherently challenging for graduate students on a strict timeline). However, when co-production is done well, it yields actionable knowledge that is readily embraced by those involved ensuring research is more broadly relevant to society (Beier *et al.,* 2017; for a review on co-production knowledge and practices please see Cooke *et al.,* 2025).

### How do we move from data to action?

Most researchers working in the conservation physiology space, regardless of their use of lab or field approaches, are doing so given their desire to generate new knowledge that can help to address the biodiversity crisis and inform conservation decisions for the benefit of wildlife and people. Given the urgency of many issues (e.g. reversing population declines and extirpations, preventing/managing disease outbreaks and spillovers, climate change adaptation strategies) and limited resources to support such work, data generated by conservation physiologists needs to have the potential to inform action (e.g. policy development, decision making about conservation interventions, regulatory decisions, etc.). While here we have illustrated the benefits of working in the liminal spaces between lab and field studies in producing impactful research outcomes, merely bridging the lab/field divide is insufficient for meaningful impacts on conservation. In short, publishing research findings alone is not enough to connect our science to policy and practice, as has been well articulated in many studies of the knowledge-action gap (e.g. Fausch *et al.,* 2002; Cook *et al.,* 2013).

So how do we move from data to action? Earlier in this paper, we introduced the concept of co-production, which has repeatedly been identified as the single most important thing a researcher can do to generate more relevant and actionable science (see Beier *et al.,* 2017 for an excellent guide). The dividends (personally, professionally, for nature and for people) derived from such actions can be immense, although the additional time investment is not insignificant (see Madliger *et al.,* 2020 for case studies of successes and failures). Collaboration between researchers and managers on projects can build trust and facilitate communication to help ensure mutually beneficial outcomes (Fig. 2). Indeed, collaborating wildlife managers can contribute to a study's questions and design to ensure that researchers understand what information is needed to support their decisions. Managers can also articulate their concerns around uncertainty in collected data, which researchers may then be able to address through modifications to an experimental design or additional research. Similarly, managers who spend time in the lab or field with the research team can gain a deeper appreciation for how the research is conducted, including its constraints and limits, which further builds trust and understanding. Ideally, co-production allows science-based knowledge to be incorporated into management decisions before academic papers are published. Increasingly, individuals trained in co-production and knowledge brokering (i.e. knowledge brokers) have particular expertise in doing such work, and funding opportunities now exist to include such experts to aid researchers in moving from data to action (Hering, 2016; Cvitanovic *et al.,* 2025). Co-production is a journey and, when entered into with good intention, humility and a willingness to learn and share power (especially when working with community members) on behalf of all actors, is itself a success.

## Conclusion

Laboratory and field-based research, as well approaches that fall somewhere in between, are all essential elements of conservation physiology. Here, we have made a case for working across the lab-field continuum as a means of developing conservation research that can better inform policy and practice (Fig. 1). Although we have focused primarily on the two ends of the spectrum (i.e. laboratory and field), we nonetheless emphasize the importance of integrative approaches (e.g. mesocosms) and technologies that provide a middle ground and offer more control than field studies yet more realism than laboratory studies. We also highlight the benefits that can arise from combining laboratory and field methods in a single study or project. Finally, we acknowledge that scientists cannot achieve meaningful conservation outcomes alone. The long-standing paradigm that ‘scientists know best’ has contributed to the knowledge-action gap and resulted in research that is ignored or dismissed by decision makers or other ‘end users’. The conservation physiology research enterprise is being re-envisioned as it becomes increasing apparent that the greatest success is achieved when collaborating with partners, ideally using a co-production model. Doing so increases the relevance of the research and increases the likelihood of generating new knowledge and understanding that is actionable. Scientists, especially in applied domains such as conservation physiology, are increasingly judged not by the impact factor of their work, but rather by the impact of their work for society. Integrating research along the entire lab-field continuum in an engaged and respectful manner with partners will yield science that can contribute towards reversing the biodiversity crisis for the benefit of wildlife and people.

## Data Availability

There is no data associated with this manuscript.
